# Long-term potentiation of glycinergic synapses by semi-natural stimulation patterns during tonotopic map refinement

**DOI:** 10.1038/s41598-020-73050-y

**Published:** 2020-10-09

**Authors:** Eva C. Bach, Karl Kandler

**Affiliations:** 1 Departments of Neurobiology, Otolaryngology, and Bioengineering, University of Pittsburgh School of Medicine, Biomedical Science Tower 3, Rm. 10016, 3501 Fifth Avenue, Pittsburgh, PA 15261 USA; 2 Present Address: Department of Physiology and Pharmacology, Wake Forest School of Medicine, PTCRC, Rm. 230, 115 South Chestnut Street, Winston-Salem, NC 27157 USA

**Keywords:** Auditory system, Neural circuits, Synaptic plasticity

## Abstract

Before the onset of hearing, cochlea-generated patterns of spontaneous spike activity drive the maturation of central auditory circuits. In the glycinergic sound localization pathway from the medial nucleus of the trapezoid body (MNTB) to the lateral superior olive (LSO) this spontaneous activity guides the strengthening and silencing of synapses which underlies tonotopic map refinement. However, the mechanisms by which patterned activity regulates synaptic refinement in the MNTB-LSO pathway are still poorly understood. To address this question, we recorded from LSO neurons in slices from prehearing mice while stimulating MNTB afferents with stimulation patterns that mimicked those present in vivo. We found that these semi-natural stimulation patterns reliably elicited a novel form of long-term potentiation (LTP) of MNTB-LSO synapses. Stimulation patterns that lacked the characteristic high-frequency (200 Hz) component of prehearing spike activity failed to elicit potentiation. LTP was calcium dependent, required the activation of both g-protein coupled GABA_B_ and metabotropic glutamate receptors and involved an increase in postsynaptic glycine receptor-mediated currents. Our results provide a possible mechanism linking spontaneous spike bursts to tonotopic map refinement and further highlight the importance of the co-release of GABA and glutamate from immature glycinergic MNTB terminals.

## Introduction

An important step in brain development is the structural and functional refinement of immature synapses and neuronal circuits to establish precisely organized and physiologically fine-tuned circuits. In the auditory system, a high degree of developmental refinement occurs not only in cortical areas^1–3^ but also in a number of subcortical nuclei including the auditory midbrain and several brainstem nuclei^4,5^. Synaptic maturation and circuit refinement have been especially well characterized in the lateral superior olive (LSO), a binaural brainstem nucleus which encodes interaural sound level differences that serve as a major cue for sound localization. LSO neurons encode interaural level differences by integrating excitatory, glutamatergic inputs from the ipsilateral cochlear nucleus, which is driven by the ipsilateral ear, with inhibitory, glycinergic inputs from the medial nucleus of the trapezoid body (MNTB), which is driven by the contralateral ear^6–8^. Both of these afferent pathways are tonotopically organized and are aligned so that individual LSO neurons are excited and inhibited by the same sound frequency^6–9^.

The high degree of precision of the tonotopic organization of the glycinergic MNTB-LSO pathway in mature animals is not present at early developmental stages but emerges gradually by processes that involve the silencing of most initial MNTB-LSO connections and the strengthening of maintained ones^10–15^. In altricial rodents, such as mice, rats, and gerbils, this refinement occurs during the first two postnatal weeks and during a time when MNTB-LSO synapses exhibit a number of transient properties such as acting as excitatory rather than inhibitory synapses due to a high intracellular chloride concentration in LSO neurons^16–20^ or the co-release of GABA and glutamate from MNTB terminals^12,21–23^. Glutamate co-release plays an important role in the tonotopic refinement of the MNTB-LSO pathway as abolishing glutamate release by genetic deletion of the vesicular glutamate transporter 3, the vesicular transporter responsible for loading vesicles in MNTB terminals with glutamate, leads to deficits in the strengthening and silencing of MNTB-LSO connections which in turn results in a lower precision of the tonotopic organization of the MNTB-LSO pathway^24^. At MNTB-LSO synapses, co-released glutamate can activate postsynaptic NMDA receptors on LSO dendrites leading to local increases in intracellular calcium^25^, which in many systems can trigger synaptic plasticity^26,27^. Very little is known about the developmental function of GABA co-release from immature glycnergic synsapses, although recent studies suggest that at the MNTB-LSO synapse, GABA does not act as a classical fast neurotransmitter but rather acts on extrasynaptic pre- and postsynaptic receptors to regulate LSO excitability^28^. In addition, co-released GABA can recruit nearby MNTB terminals by acting on presynaptic, depolarizing GABA_A_ receptors, a mechanism that may contribute to the co-activation of similarly-tuned MNTB inputs^23^.

Tonotopic refinement of the MNTB-LSO pathway by synaptic silencing and strengthening occurs before hearing onset and thus in the absence of sound-elicited activity^11,14,15,29^. Nevertheless, this refinement depends on temporally structured spontaneous spike activity, which is generated in the immature cochlea from where it is propagated along the central auditory pathway^14,30–33^. Eliminating or changing the natural pattern of prehearing spike activity leads to a disruption of the maturation of auditory synapses and neurons, a deficit in the tonotopic refinement of the MNTB-LSO pathway^13–15,34,35^, and impairments in central auditory processing^36^. Despite its important role in the maturation of central auditory circuits, the synaptic and cellular mechanism by which patterned spike activity mediates the refinement of tonotopic maps has remained poorly understood.

Activity-dependent changes in synaptic strength such as long-term potentiation (LTP) and long-term depression (LTD) are cellular correlates of learning and memory and also play important roles in the developmental refinement of neuronal circuits^37–40^. The mechanisms of LTP have been extensively investigated for glutamatergic and GABAergic synapses^41,42^ but much less is known about activity-dependent plasticity at glycinergic synapses^43–45^. In the auditory brainstem, glycinergic LTD is expressed at immature MNTB-LSO synapses before hearing onset^46,47^ and glycinergic LTP is expressed in the medial superior olive (MSO) and LSO after the onset of hearing, when MNTB-LSO synapses have acquired most of their mature properties^48,49^. In both MSO and LSO, the induction of LTP after hearing onset depends on a co-activation of glutamate receptors, which in vivo could be achieved by sound-driven activation of converging ipsilateral glutamatergic and contralateral glycinergic pathways. However, such temporal correlation of glycinergic and glutamatergic inputs is unlikely to occur before hearing onset because spontaneous activity is not coordinated between the two cochlea^50^. This raises the question whether and what forms of LTP are expressed in the LSO before hearing onset, the developmental period when the multi-fold strengthening of MNTB-LSO connections actually occurs^11,12,14,15,29,51^.

In this study we addressed this question by exploring the stimulus conditions that are able to induce LTP at MNTB-LSO synapses and the mechanisms that mediate its induction and expression. Using whole-cell recordings from LSO neurons in brainstem slices from one-week-old mice, we found that stimulus patterns that mimic the spontaneous patterns in vivo at this age are highly effective in inducing a novel form of glycinergic LTP. This form of LTP is calcium dependent, requires the cooperative activation of GABA_B_ and metabotropic glutamate receptors, and resulted in an increase in postsynaptic glycine receptor-mediated currents. Thus, the co-release of GABA and glutamate from immature MNTB terminals enables the expression glycinergic LTP at an age before sound can elicit coordinated activity in excitatory and inhibitory afferent synapses to the LSO.

## Results

### Semi-natural stimulation patterns are effective in inducing LTP in the MNTB-LSO pathway

**Figure 1:**
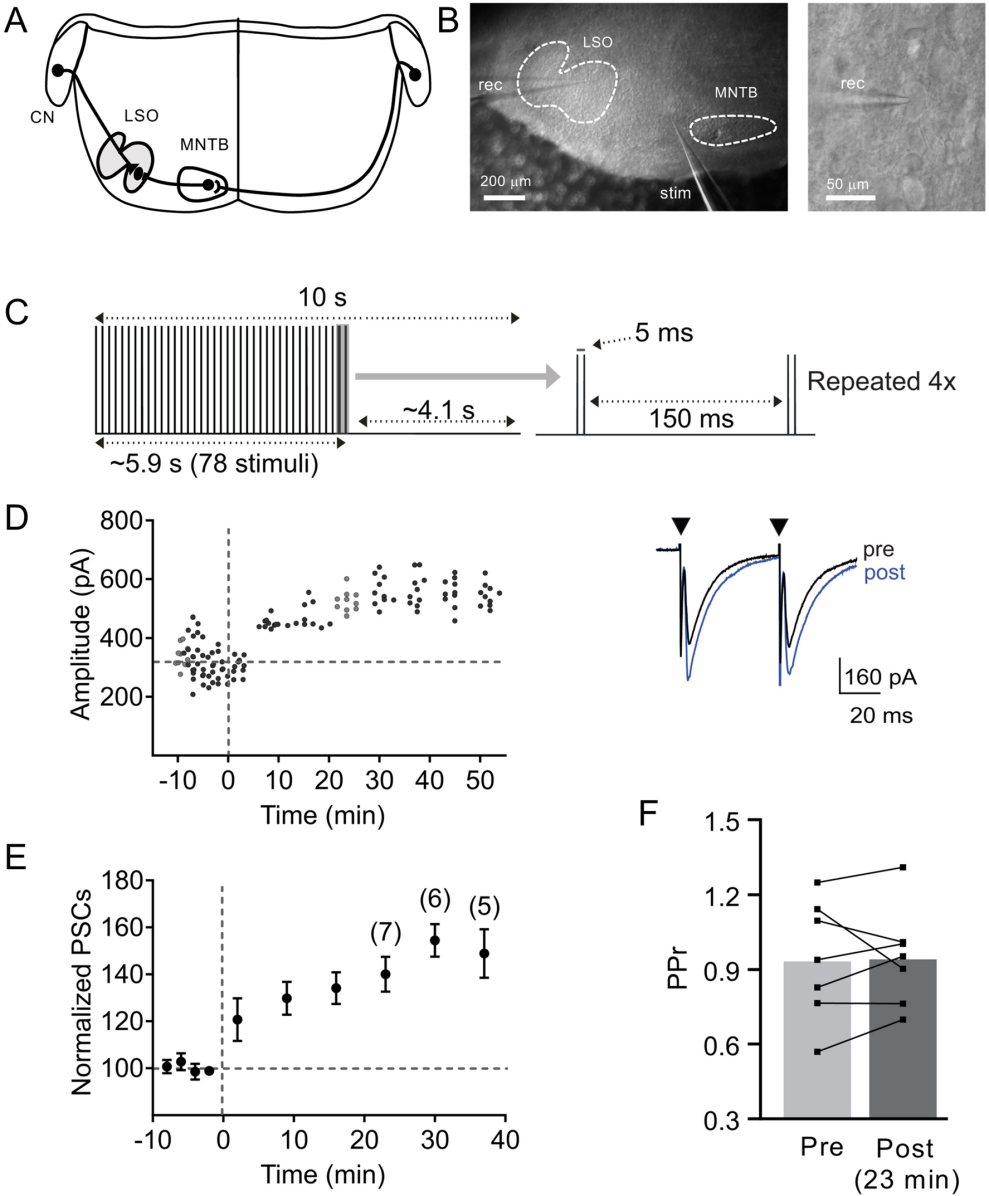
SNP stimulation elicits LTP in the MNTB-LSO pathway. (**A**) Schematic illustration of the afferent inputs to LSO. (**B**) Photomicrographs from a brainstem slice containing the MNTB and LSO illustrating the position of the stimulation (stim) and recording (ref) electrodes. (**C**) Schematic illustration of the SNP stimulus paradigm. (**D**) MNTB-evoked response amplitudes of a neuron increase after SNP stimulation. Traces to the right depict average responses (ISI 50 ms) during baseline (black) and at 23 min after SNP stimulation (blue). (**E**) Normalized population responses. Numbers in parenthesis indicate number of neurons recorded until and after 23 min post induction. (**F**) Individual neuron (connected lines) and average group (bars) PPRs prior to and 23 min post SNP stimulation. Normalized population responses and PPRs are presented as mean ± SEM.

Recordings were performed in slices from mice aged postnatal day 5–8, an age when MNTB-LSO synapses are strengthened in vivo^11,12,14,15,29,51^. At this age, the activity of MNTB neurons in vivo reflects the pattern of cochlear generated activity, which consists of periodic bouts of action potentials that occur about 5 times per minute. During these activity bouts, action potentials are clustered together in ‘mini bursts’ that are separated by about 150 ms and contain 2–4 spikes at around 200 Hz^14,30–32^. To explore the effects of these stereotypical spike patterns on immature MNTB-LSO synapses, we tested whether stimulating MNTB-LSO connections in vitro (Fig. 1A,B) with a pattern that mimicked the in vivo pattern changes the strength of these connections. These semi-natural stimulation patterns (SNPs) consisted of 4 bouts of electrical stimuli, each of which contained 78 stimuli that were grouped in pairs (“mini-bursts”, 5 ms inter-stimulus interval) and separated by 150 ms (Fig. 1C). Stimulation of the MNTB-LSO pathways with SNPs elicited a potentiation of MNTB-LSO synapses in 86% of neurons (6 out of 7 neurons). The potentiation was long-lasting and on average increased the peak amplitude of responses to 140.0 ± 7.4% of baseline amplitudes (measured at ~ 23 min post induction, n = 7, *p* < 0.001, two-tailed paired t-test, Fig. 1D,E).

Synaptic potentiation can result from postsynaptic and/or presynaptic mechanisms, the latter of which involves an increase in neurotransmitter release due to an increase in the probability of release^52,53^. A change in the probability of release is expected to decrease the paired pulse ratio (PPR), which is defined as the ratio of the amplitude of the second response to two stimuli to the amplitude of the first response. In accordance with previous studies at this age^12,47^, the PPR of MNTB-LSO synapses before potentiation was close to 1 (0.94 ± 0.09, n = 7) and most importantly was not changed by synaptic potentiation (0.95 ± 0.07, n = 7, *p* = 0.949, two-tailed paired t-test, Fig. 1F). This suggests that SNP-induced LTP at MNTB-LSO synapses is primarily due to changes in the postsynaptic neuron.

### Tetanic or low-frequency stimulation does not induce LTP in the MNTB-LSO pathway

**Figure 2:**
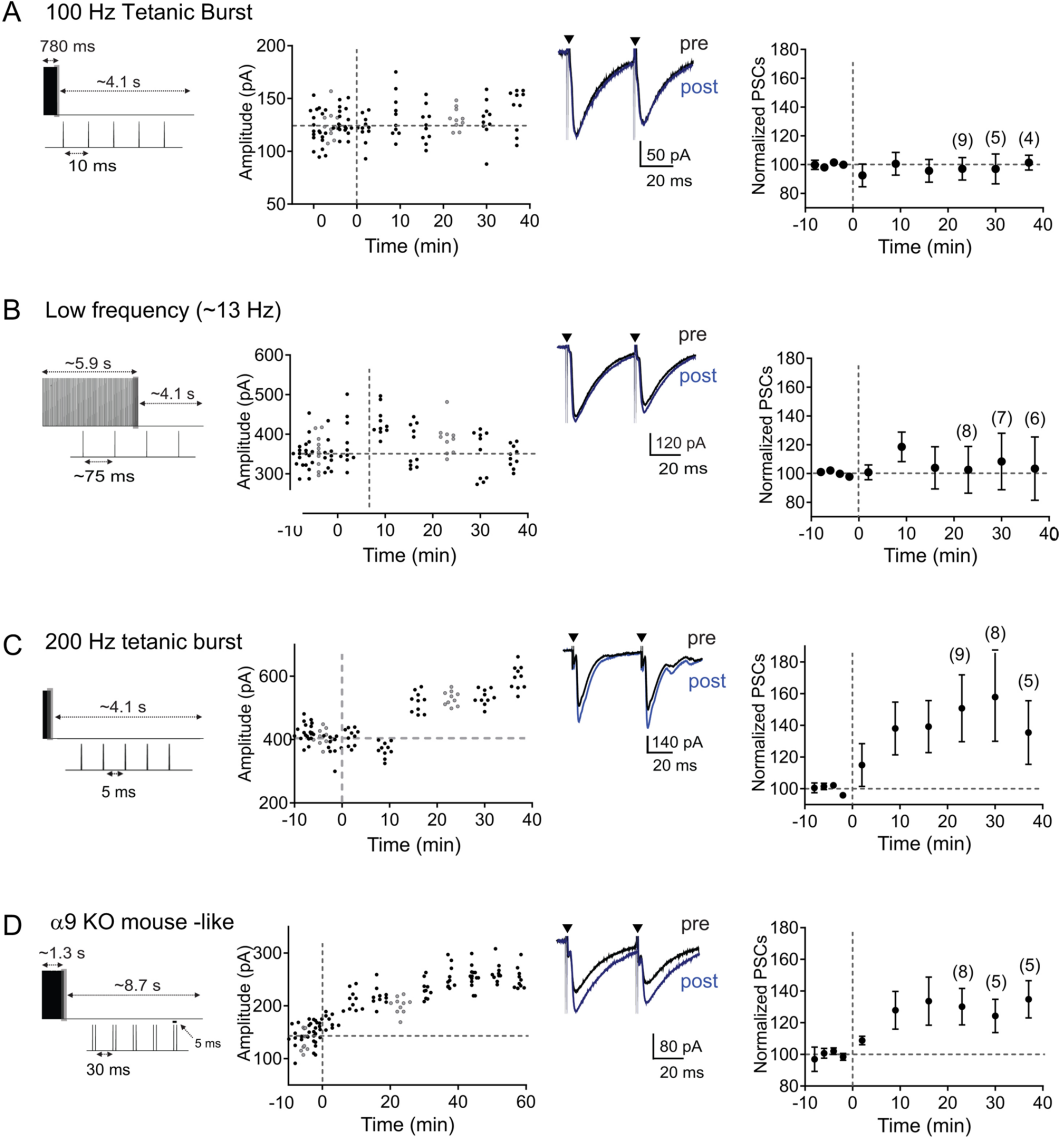
The temporal structure of stimulation patterns is critical for the induction of LTP in the MNTB-LSO pathway. Left column: schematic diagrams of induction stimulus paradigms (**A**) 100 Hz tetanic burst, (**B**) low frequency stimulation, (**C**) 200 Hz tetanic burst, and (**D**) simulated α9-KO mouse pattern. Middle column shows example cells with traces indicating averaged responses during baseline (black) and at 23 min after induction stimuli (blue). Right column shows normalized population responses. Numbers in parenthesis indicate number of neurons recorded until and after 23 min post induction. Normalized population responses are presented as mean ± SEM.

To explore the significance of the temporal structure of SNP in inducing LTP, we applied the same number of electrical stimuli and bursts but changed their interspike intervals (ISIs) (Fig. 2). Because tetanic stimulation (100 Hz) reliably elicits LTP in many systems^54–56^, we first tested whether delivering the same number of stimuli within a burst at 100 Hz, while maintaining an inter-burst interval of ~ 4.1 s, could elicit LTP (Fig. 2A). However, following tetanic burst stimulation, we observed no change in the PSC amplitudes (97.0 ± 7.8% of baseline PSC amplitude n = 9, *p* = 0.711, two-tailed paired t-test, Fig. 2A). This result indicates that temporal components of the SNP pattern, omitted in the 100 Hz tetanus, are essential for LTP in the MNTB-LSO pathway.

We next tested whether a burst pattern which omitted the two distinct ISIs (5 and 150 ms) present in the SNPs can elicit LTP. In this induction protocol we maintained the overall burst duration (~ 5.9), stimulus number (78) and inter-burst interval (~ 4.1 s) but spaced the stimuli inside a burst equally with a 76 ms ISI (Fig. 2B). Stimulation with this low-frequency protocol also failed to induce LTP (102.6 ± 16.2% of baseline PSC, n = 8, *p* = 0.876, two-tailed paired t-test, Fig. 2B) suggesting that one or both of the principal ISIs that are present in SNPs carry cues necessary for LTP induction.

### Inter-stimulus intervals of 5 ms are necessary and sufficient to elicit LTP in the MNTB-LSO pathway

To further explore the significance of the within-burst ISIs in LTP induction we tested whether an ISI of 5 ms is sufficient to elicit LTP by delivering a burst of 78 stimuli at 200 Hz at a burst interval of ~ 4.1 s. This stimulus maintained most of the features of the SNP stimulation, with the exception of the 150 ms ISI between ‘mini-bursts’. Applying this protocol reliably elicited LTP (150.8 ± 21.1%, n = 9, *p* = 0.029, two-tailed paired t-test, Fig. 2C) with a similar average magnitude as observed after SNP application (SNP LTP 140.0 ± 7.4%; difference between SNP- and 200 Hz- LTP, measured at 23 min post induction: *p* = 0.674, two-tailed unpaired t-test) and in a similar number of neurons tested (86% in response to SNP and 78% for the 200 Hz stimulus).

Previous studies demonstrated that mice that lack the α9 subunit of nicotinic acetylcholine receptors and as a result, lack functional nicotinic cholinergic transmission to cochlear hair cells (α9 KO mice^57^) have abnormal patterns of cochlea-generated spontaneous activity before hearing onset and an impaired developmental strengthening of MNTB-LSO connections^14^. To test whether the deficits in synaptic strengthening in α9 KO mice may result from deficits of this altered activity pattern to induce LTP, we stimulated the MNTB-LSO pathway using activity patterns mimicking those previously recorded in vivo in 8-day old α9 KO mice^14^. In particular, α9 KO mice have unchanged average activity levels but mini-bursts during an activity bout occur at shorter intervals, resulting in shorter overall burst duration and prolonged inter-burst intervals. To recapitulate this pattern in vitro, we stimulated the MNTB-LSO pathway with four stimuli bursts, 8.7 s apart, each of which contained 78 stimuli with a 5 ms ISI between the two stimuli of a mini-burst and a 30 ms ISI between mini-bursts (Fig. 2D). These α9 KO-like stimulus protocols lead to a significant increase of the PSC amplitude to an average of 130.1 ± 11.6% (n = 8, *p* = 0.021, two-tailed paired t-test, Fig. 2D), which was not significantly different from the magnitude of LTP observed after SNP stimulation (140.0 ± 7.4%, *p* = 0.498 between SNP- and α9- LTP, two-tailed unpaired t-test). This result indicates that the precise inter-burst interval is not crucial for the induction of LTP and further highlights the significance of stimuli with an ISI of 5 ms. In addition, they imply that the refinement deficits in α9 KO-mice are unlikely due to failure of their altered activity patterns to induce LTP in the MNTB-LSO pathway.

### LTP induction does not require postsynaptic depolarizations or action potentials

**Figure 3:**
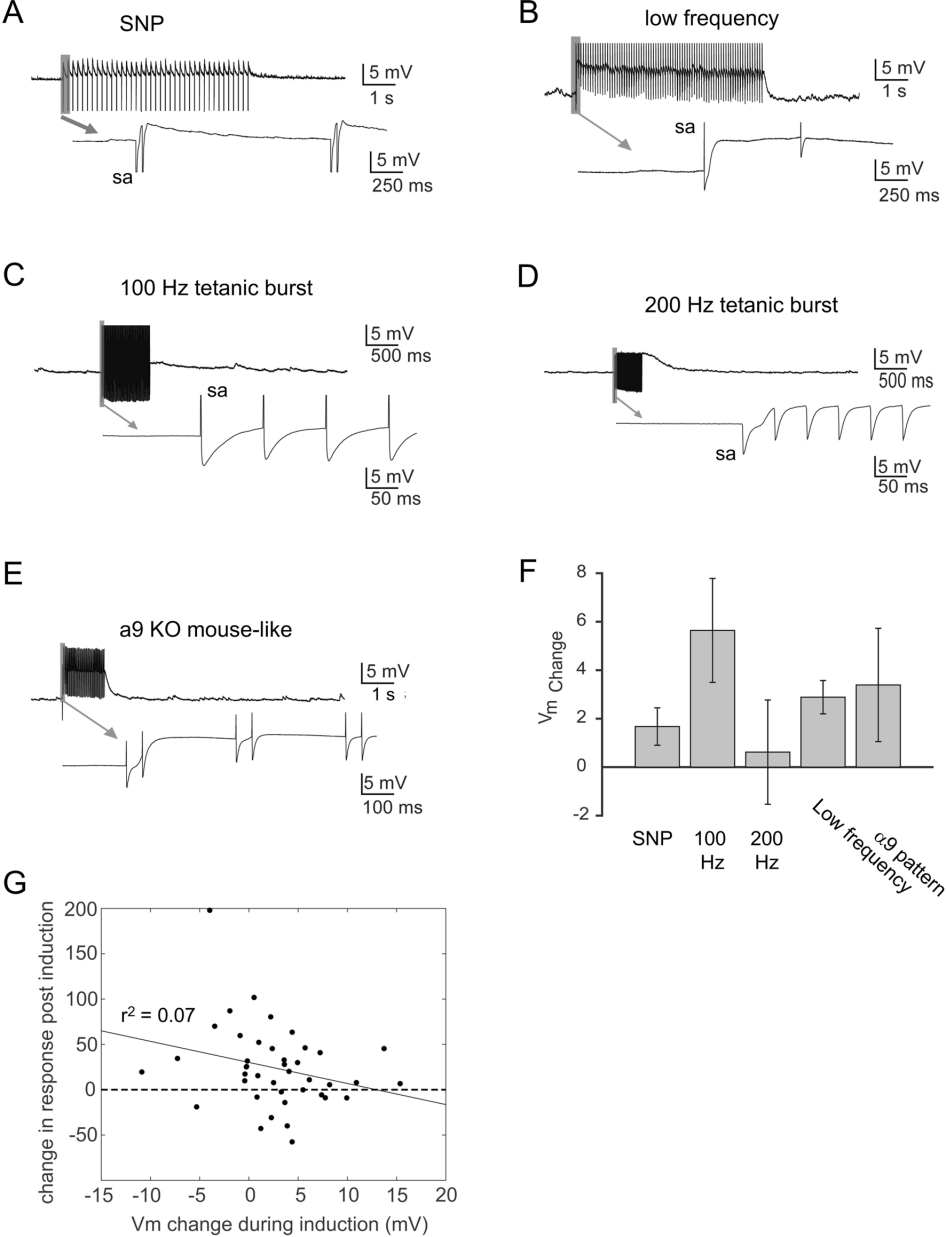
Postsynaptic membrane potential responses during induction stimulation. (**A**–**D**) Responses during one of four delivered stimulus bursts. Example cells correspond to the neurons shown in Figs. 1 and 2. (**F**) Average of membrane potential change for each stimulation group during stimulus induction. Data are presented as mean group membrane potential change ± SEM. (**G**) The direction or magnitude of membrane potential responses showed no correlation with the change in responses measured 23 min following induction.

Both in the MSO and LSO, induction of LTP after hearing onset requires coincident postsynaptic depolarizations^48,49^. In our experiments we did not expect that MNTB stimulation elicits significant postsynaptic depolarizations because we used a pipette chloride concentration (10 mM) with an estimated chloride reversal potential of − 66 mV, which is near the resting membrane potential and approximately matches the average native intracellular chloride concentration in LSO neurons at that age^16–18,58^. However, under high-frequency stimulation, hyperpolarizing GABAergic responses can become depolarizing due to a breakdown of the transmembrane chloride gradient and the generation of a bicarbonate mediated outward current^59^. This raises the possibility that the high frequency activity that is present in some of our induction stimulation protocols may lead to strong or even suprathreshold responses, which in turn may enable LTP induction. Analysis of synaptic membrane potential responses during induction protocols shows that most LSO neurons generated small, subthreshold depolarizations (1–5 mV from a Vrest of − 68.4 ± 0.7 mV) which could temporally summate to a plateau that lasted throughout the stimulation burst (Fig. 3). In only a small percentage of neurons (5 of 41) did these depolarizations trigger action potentials. Importantly, the direction and magnitude of postsynaptic depolarizations did not significantly differ between stimulus patterns (Fig. 3F; V_m_ change: SNP: 1.7 ± 0.8 mV, n = 7; 100 Hz: 5.6 ± 2.0 mV, n = 9; 200 Hz: 0.6 ± 2.3 mV, n = 9; low frequency: 2.9 ± 0.7 mV, n = 8; α9 pattern: 3.39 ± 1.92 mV, n = 8; *p* = 0.363; 1-way ANOVA). In addition, we found no correlation between the direction and magnitude of synaptic responses during induction and the presence or the magnitude of LTP (Fig. 3G). These results indicate that induction of LTP before hearing onset does not depend on postsynaptic membrane depolarizations or action potentials.

### Induction of LTP in the MNTB-LSO is calcium-dependent but NMDA receptor-independent

**Figure 4:**
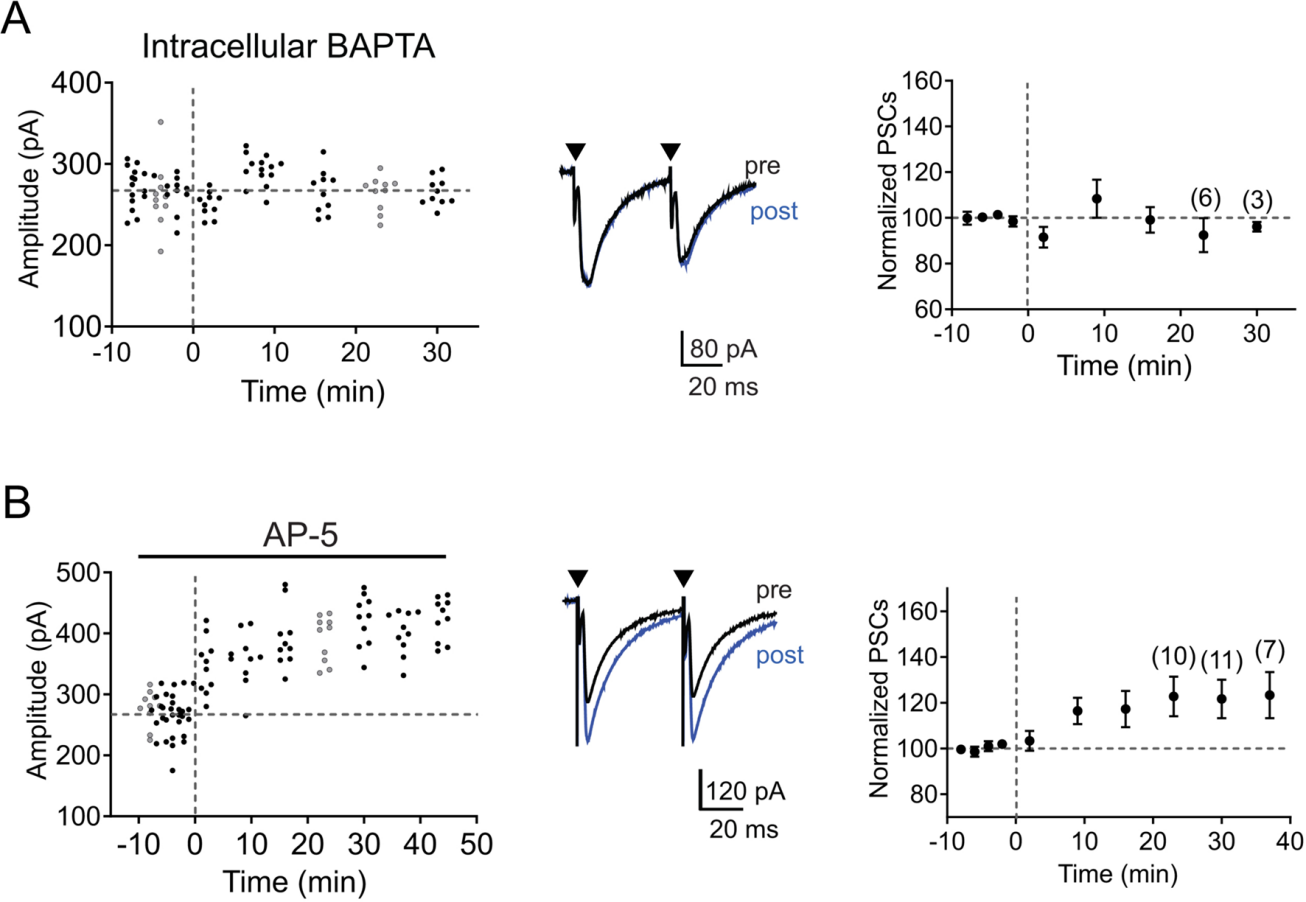
LTP in the MNTB-LSO pathway is Ca^2+^ dependent, but NMDA receptor independent. (**A**) and (**B**) Time course of response amplitudes in an individual neuron (left) and from the population of neurons (right) in response to SNP stimulation (dashed line) with (**A**) intracellular BAPTA (20 mM) and (**B**) bath applied AP-5 (100 μM). Normalized population responses are presented as mean ± SEM.

Essentially all forms of synaptic potentiation described so far require an initial increase in the intracellular Ca^2+^ concentration^41,60^. To test whether this also holds true for LTP in the MNTB-LSO pathway, we chelated intracellular Ca^2+^ by including the Ca^2+^ chelator BAPTA (20 mM) in the recording pipette solution. LSO neurons perfused with BAPTA failed to exhibit LTP in response to the SNP stimulation (92.5 ± 7.5%, n = 6, *p* = 0.337, two-tailed paired t-test, Fig. 4A) indicating that LTP is calcium-depended. To address how MNTB inputs increase postsynaptic calcium concentrations, we next tested whether NMDA receptors, which are responsible for inducing LTP in a large variety of synapses, including glycinergic synapses in the MSO^49^, mediate LTP-inducing calcium influx. In developing MNTB-LSO synapses, NMDA receptors are activated by the co-release of glutamate before hearing onset, and induce a local rise in the dendritic intracellular calcium concentration without accompanying noticeable membrane depolarizations^22,25^. However, these dendritic NMDA receptor-mediated calcium responses are not necessary for induction of LTP because blocking NMDA receptors by bath application of the NMDA receptor antagonist AP-5 (100 µM) had no effect on LTP (Fig. 4B). The magnitude of LTP by SNP stimulation during application of AP-5 (122.8 ± 8.7% of baseline PSC amplitudes, n = 11, *p* = 0.016, two-tailed paired t-test, Fig. 4B), was not significantly different from the magnitude of LTP under control conditions (*p* = 0.186, two-tailed unpaired t-test, compare Fig. 1B,C with Fig. 4B).

### LTP at MNTB-LSO synapses requires activation of g-proteins by mGlu and GAGA_B_ receptors acting cooperatively

**Figure 5:**
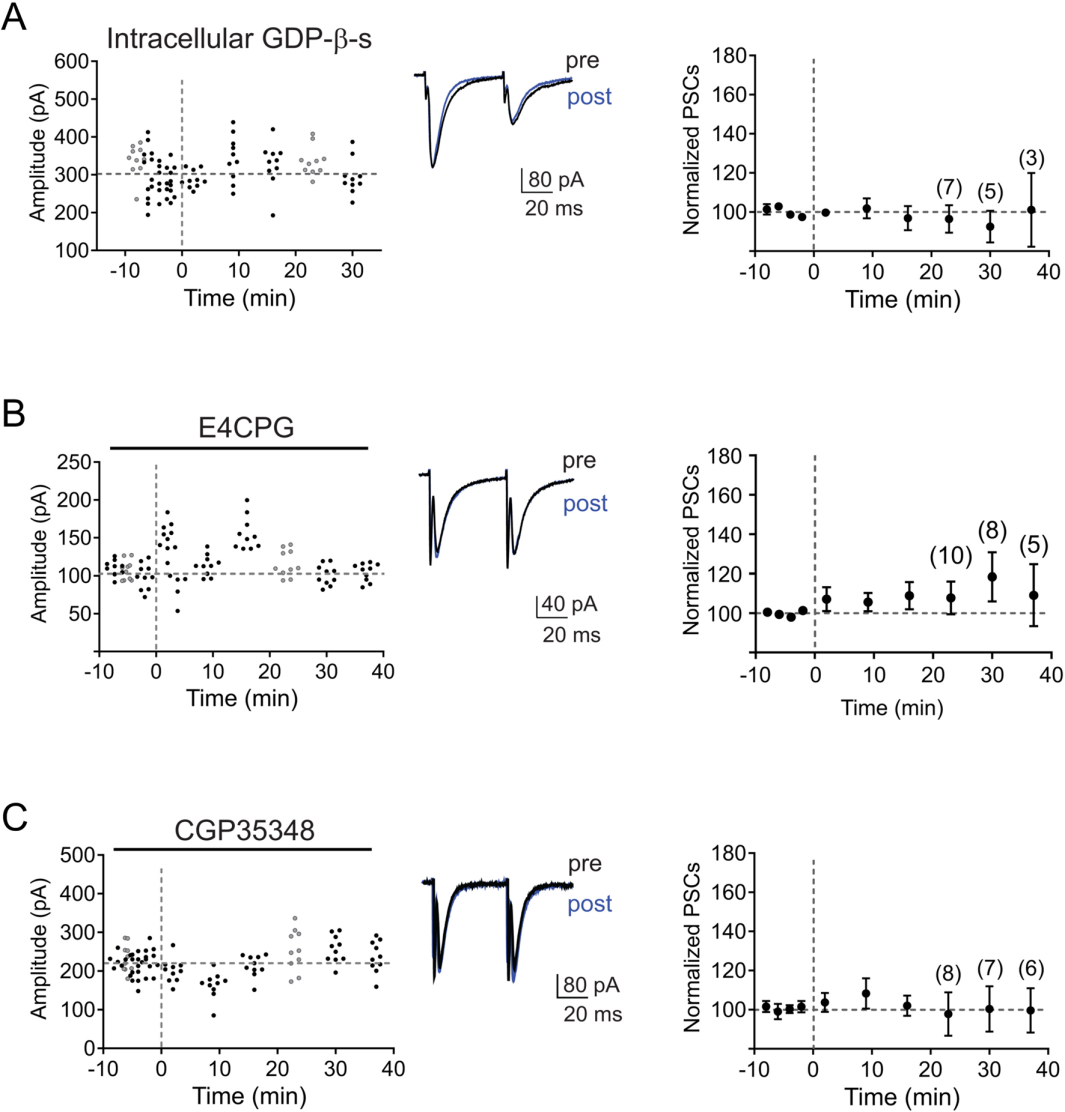
LTP in the MNTB-LSO pathway requires activation of g-proteins and metabotropic receptors. Time course of response amplitudes in individual neurons (left) and the population of neurons (right) in response to SNP stimulation (dashed lines). Traces to the right depict average responses (ISI 50 ms) during baseline (black) and at 23 min after SNP stimulation (blue). (**A**) Responses with intracellular perfusion of GDP-β-s (2 mM), (**B**) bath application of the mGluR antagonist E4CPG (500 μM), and (**C**) bath application of the GABA_B_ receptor antagonist CGP35348. Normalized population responses are presented as mean ± SEM.

Having ruled out the requirement of NMDA receptor-mediated Ca^2+^ influx for the induction of LTP, we next tested the contribution of g-protein intracellular signaling that can lead to the release of Ca^2+^ from intracellular stores. In a first step, we blocked all g-protein-dependent signalling by substituting GTP in the recording electrode solution with the broad-spectrum g-protein blocker GDP-β-s (2 mM)^61,62^. In the presence of GDP-β-s we observed no significant change in PSC amplitudes in response to SNP stimulation (96.5 ± 7.0% of baseline PSC amplitude, n = 7, *p* = 0.622, two-tailed paired t-test, Fig. 5A), indicating that LTP depends on the postsynaptic activation of g-protein coupled receptors. Due to their co-release of glutamate and GABA^12,21,22,28,63^, MNTB terminals could activate g-protein coupled metabotropic glutamate receptors (mGluRs) or GABA_B_ receptors, both of which are expressed in LSO neurons before hearing onset^28,64–67^. Since activation of group I or group II mGluRs in immature LSO neurons by high-frequency stimulation of glutamatergic synapses elicit long-lasting calcium responses^64,65^, we first tested whether mGluRs are necessary for LTP induction by blocking mGluRs with the group I and II mGluR antagonist E4CPG (500 µM). Under this condition, SNP failed to induce LTP (post induction PSC amplitude 107.7 ± 8.3% of baseline, n = 9, *p* = 0.366, two-tailed paired t-test, Fig. 5B) indicating that MNTB-LSO stimulation can activate group I and II mGluRs and that this activation is necessary for inducing LTP. Next, we investigated the possible contribution of GABA_B_ receptors, who mediate LTD at MNTB-LSO synapses^67^ and in hippocampal pyramidal neurons, act cooperatively with mGluRs to induce LTP at GABAergic synapses^61^. When we applied SNP stimulation in the presence of the specific GABA_B_ receptor antagonist CGP35348 (100 µM) we observed no LTP (PSC amplitudes 97.8 ± 11.1% of baseline, n = 8, *p* = 0.848, two-tailed paired t-test, Fig. 5C) confirming that MNTB-LSO terminals can activate GABA_B_ receptors^21,28^ and that GABA_B_ receptor activation is necessary for the induction of LTP. In summary, our results establish the necessity of a cooperative activity of GABA_B_ and mGluR-dependent g-protein-mediated signaling in inducing glycinergic LTP at MNTB-LSO synapses.

### LTP involves an increase in glycine receptor-mediated responses

**Figure 6:**
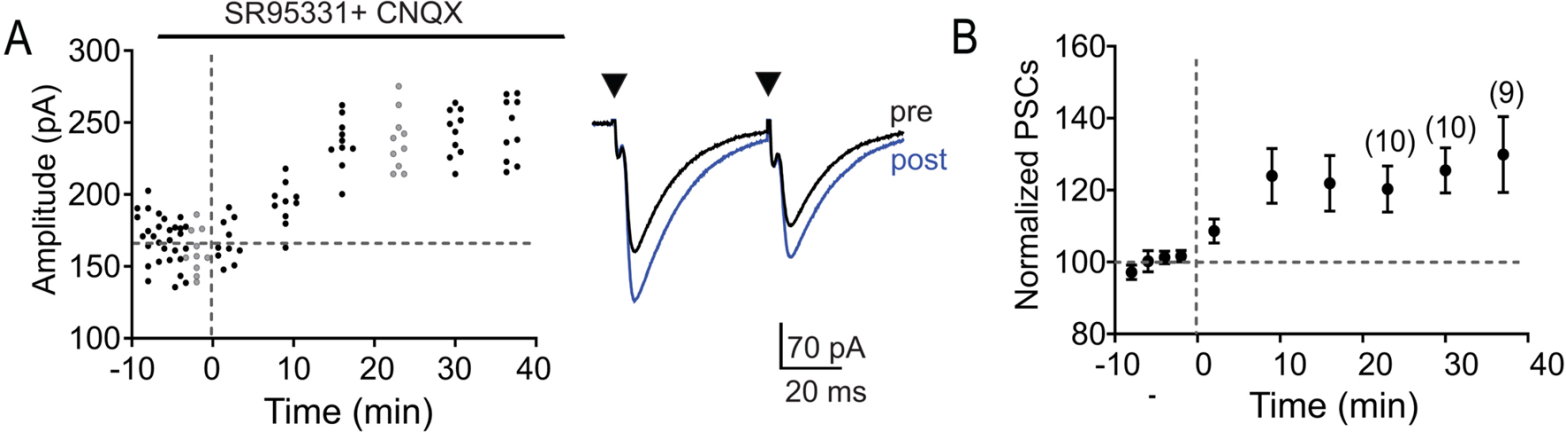
LTP in the MNTB-LSO pathway potentiates glycine receptor mediated conductance in the presence of SR95331 (20 μM) and CNQX (20 μM). (**A**) Time course of response amplitudes in an individual neuron. Traces depict average responses (ISI 50 ms) during baseline (black) and at 23 min after SNP stimulation (blue). (**B**) Normalized population responses. Numbers in parenthesis indicate number of neurons recorded until and after 23 min post induction. Normalized population responses are presented as mean ± SEM.

LTP did not change PPR and therefore most likely is expressed by postsynaptic changes (Fig. 1D). Because before hearing onset MNTB-LSO synapses co-release glycine, GABA and glutamate, LTP could involve an increase in currents mediate by glycine, GABA_A_, or AMPA receptors. To examine these possibilities, we isolated glycine receptor-mediated responses by bath application of the AMPA receptor antagonist CNQX (20 µM) and the GABA_A_ receptor antagonist SR 95,531 (20 µM). Due to the voltage-dependent block of NMDA receptors at our negative holding potentials (V_hold_ − 84 mV) during test responses and due to the very small amplitude of NMDA receptor mediated currents with somatic recordings^22^, NMDARs are highly unlikely to contribute significantly to EPSCs under these conditions. SNP stimulation under these conditions induced LTP (PSC amplitude 120.3 ± 6.4% of baseline, n = 10, *p* = 0.005, two-tailed paired t-test, Fig. 6), with a magnitude that was not significantly different from that induced in control conditions (*p* = 0.065, two-tailed unpaired t-test, compare Fig. 6 with Fig. 1B,C). In a separate group of neurons, we also tested the response to SNP stimulation in the presence of CNQX alone. In these neurons, we also observed LTP as indicated by an increase of responses to 135.4 ± 7.8% of baseline (n = 5, *p* = 0.002, paired t-test), which was not significantly different to the magnitude of LTP in control experiments (*p* = 0.681, two-tailed unpaired t-test). Taken together, these results indicate that LTP in the MNTB-LSO pathway potentiates glycine receptor-mediated currents. Due to the very small amplitude of isolated GABA_A_ receptor-mediated currents, we were unable to reliably evaluate their contribution to the overall magnitude of potentiation, leaving open the possibility that the potentiation of GABA_A_ receptor-mediated responses contribute to LTP to a minor degree.

## Discussion

In this study we demonstrate that semi-natural stimulation patterns that mimic the temporal structure of spontaneous activity in vivo before hearing onset elicit a novel form of inhibitory glycinergic LTP in the developing MNTB-LSO pathway in vitro. The induction of this LTP did not require postsynaptic membrane depolarization or action potentials, but depended on an increase in the postsynaptic Ca^2+^ concentration and the activation of g-proteins through a cooperative action of mGluR and GABA_B_ receptors. LTP seems to be expressed postsynaptically as it was not associated with a change in the PPR. The properties of this inhibitory LTP make it a plausible mechanism for activity-dependent strengthening of MNTB-LSO connections and tonotopic refinement before the onset of sound driven activity.

### Semi-natural temporal patterns elicit LTP

Before hearing onset, the topographic precision of the MNTB-LSO pathway is increased by the silencing of most initial connections and the strengthening of maintained ones^11–13,15^. This synaptic remodeling critically depends on patterned spontaneous spike activity that originates in immature cochlear hair cells from which it is faithfully transmitted along the ascending auditory pathway^14,30,32^. In this study we demonstrate that stimulation of the MNTB-LSO pathway with a temporally structured stimulation pattern that reproduces the main temporal features of the spontaneous activity pattern in vivo*,* reliably elicits LTP (Fig. 1), which could be a plausible synaptic mechanism by which patterned activity contributes to the maturation and tonotopic refinement of the MNTB-LSO pathway. Although the magnitude of LTP that we observed in our acute experiments is far from the magnitude of the developmental increase in the strength of MNTB-LSO connection occurring in vivo, in many other systems LTP can lead to long-lasting functional and structural changes, which include the insertion of postsynaptic receptors^68–70^ and the formation of new synapses^71–73^. It is thus at least conceivable that such structural changes may also be triggered by glycinergic LTP and lead to the increase in the number of release sites and quantal amplitudes that underlie the 8–12 fold strengthening of single MNTB-LSO connections before hearing onset^12^.

Our results also reveal that stimulation at 200 Hz, the frequency of action potentials during mini-bursts in vivo^14,32^, is necessary and sufficient to induce LTP. 200 Hz stimuli presented as mini-bursts (Figs. 1, 2D) or as a continuous train (2C), induced LTP, while stimulation patterns that omitted stimulus intervals of 5 ms failed to induce LTP (Fig. 2A,B). Interestingly, 200 Hz stimulation is also highly effective in inducing LTP in MNTB axon collateral synapses on MSO neurons after hearing onset^49^. A sensitivity to 200 Hz (theta burst) stimulation was also reported for GABA-LTP in adult CA1 pyramidal cells, a form of inhibitory LTP that depends on the co-activation of GABA_B_ and mGluR receptors^61^ by neurotransmitter spillover^74–76^. It is likely that neurotransmitter spillover also underlies the activation of GABA_B_ and mGluRs for triggering LTP of MNTB-LSO synapses. In support of this, previous studies of immature MNTB-LSO synapses demonstrated GABA spillover to extrasynaptic GABA_A_ receptors^28^ and glutamate spillover to extrasynaptic NMDARs^77^. Along the same lines, high-frequency, but not low-frequency stimulation, of excitatory inputs elicits mGluR-mediated calcium responses in immature LSO neurons, consistent with the idea of extrasynaptic glutamate spillover to mGluRs^65^.

### Synaptic mechanisms mediating MNTB-LSO LTP

Inhibitory synapses across the central nervous systems can express a wide variety of activity-dependent plasticity with diverse stimulation requirements and mediated by distinct induction and expression mechanisms^45,78–81^. For glycinergic synapses formed by MNTB axons, previous studies demonstrated two distinct forms of age-dependent LTP in the MSO^49^ and LSO^48^, which occur after the onset of hearing. Similar to LTP reported in this study, these forms of inhibitory LTP are expressed postsynaptically and involve an increase in GlyR mediated currents. However, they differ from LTP before hearing onset in respect to their stimulation requirements as well as their induction and expression mechanisms. LTP in the MSO of gerbils after hearing depends on the activation of dendritic NMDA receptors and LTP at MNTB-LSO synapses after hearing onset is induced by low-frequency stimulation combined with glutamate-mediated postsynaptic depolarizations and GABA_B_ receptor activation^48^. Thus, after hearing onset, LTP in MNTB pathways requires coincident activity of MNTB inputs with glutamatergic inputs from the cochlea nucleus, which in vivo would occur under acoustic stimulation. In contrast, LTP in the LSO before hearing onset, despite also being dependent on glutamate release, can occur independently from cochlear nucleus inputs due to the transient co-release of glutamate from MNTB terminals that is present during this age^22^. Thus, a possible developmental function of co-release of glutamate may be to enable LTP in the MNTB-LSO pathway at an early age when cochlear-elicited activity between both sides is not yet coordinated by sound^50^, yet when the MNTB-LSO pathway is remodeled by spontaneous activity. Interestingly, mice that have a genetic loss of the vesicular glutamate transporter 3 and, as a consequence, lack glutamate release from inner hair cells and MNTB terminals^24,82^, exhibit an impaired strengthening of MNTB-LSO connection before hearing onset. This impaired strengthening may reflect deficits in LTP due to the loss of mGluR activation by MNTB terminals and/or may be due to the absence of 5 ms inter-spike intervals in the spontaneous auditory nerve activity in these mice before hearing onset^83^. Further investigations of LTP or LTD in mouse models lacking glutamate release or having altered spontaneous activity patterns can address these open questions.

**Figure 7:**
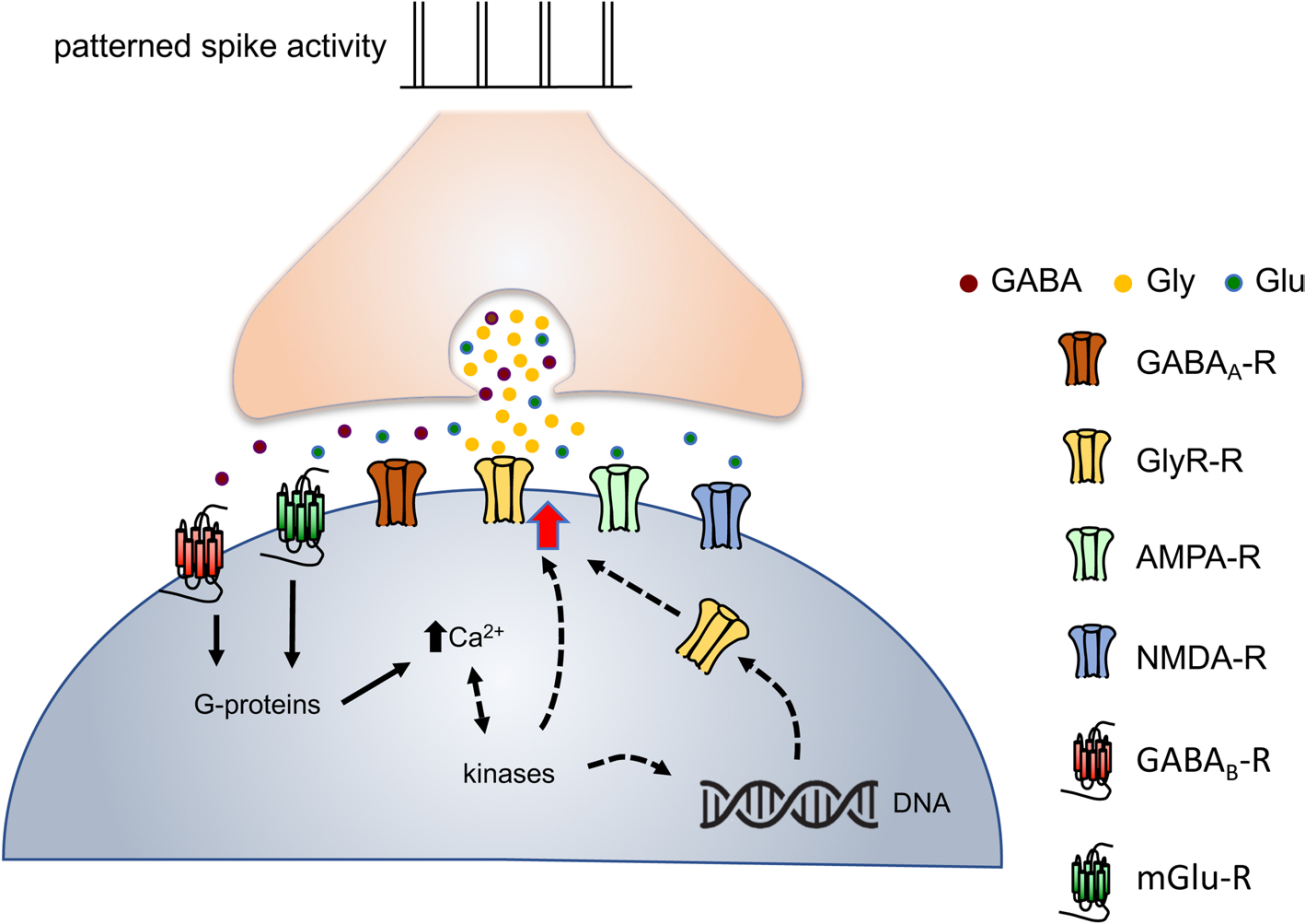
Proposed model of LTP at developing MNTB-LSO synapses. Patterns of high-frequency, bust-like spike activity lead to the activation of perisynaptic mGlu receptors and GABA_B_ receptors, which activate g-protein-dependent signaling that results in an increase in the intracellular calcium concentration. LTP is expressed postsynaptically and is characterized by an increase in glycine receptor-mediated currents, which may result from a modification of existing glycine receptors or the synaptic insertion of new receptors.

In addition to the activation of mGluR receptors, LTP at MNTB-LSO synapses requires the activation of postsynaptic GABA_B_ receptors (Fig. 7), a feature it shares with all other known forms of activity-dependent long-term plasticity at these synapses^48,67^ and with LTP of GABAergic synapses in the cortex^78^ and hippocampus^61,84^. Although postsynaptic GABA_B_ receptors are best known for their g-protein-mediated activation of potassium channels (GIRK channels), GABA_B_ receptors also have been linked to important signaling pathways that are tied to inducing synaptic plasticity^85^. For example, in cortical, cerebellar and hippocampal neurons, activation of GABA_B_ receptors can lead to the activation of phospholipase C (PLC), the generation of IP3, and the release of Ca^2+^ from intracellular stores^78,86–88^. PLC activation and an increase in intracellular Ca^2+^ concentration has been shown to be necessary for GABA_B_ receptor-dependent GABA-LTP in cortical neurons^78^ as well as for LTD at MNTB-LSO synapses^46,89^. Although previous imaging studies in the developing LSO did not detect GABA_B_ receptor-elicited Ca^2+^ responses^66^, these studies imaged only cell bodies, leaving the possibility open that GABA_B_-mediated Ca^2+^ responses occur in LSO dendrites, perhaps reflecting different effects of somatic and dendritic GABA_B_ receptors^90^. In the developing hippocampus, GABA_B_ receptor activation can also lead to a Ca^2+^-dependent release of BDNF, which in turn promotes the membrane expression of GABA_A_ receptors^91,92^. Because BDNF is also expressed in the developing LSO^93^ and is necessary for inducing GABA_B_ receptor-mediated LTD of MNTB-LSO synapses^67^, it may also participate in the induction of LTP. Finally, another possibility is that GABA_B_ receptors contribute to LTP induction by augmenting mGluR-mediated signaling^94,95^. In this scenario, one would expect that stronger activation of mGluRs, perhaps by the coincident activation of glutamatergic synapses that occurs after hearing onset, would make LTP GABA_B_ independent, increase its magnitude, or widen the spectrum of stimulation patterns that can induce it.

Although the exact signaling cascades that link GABA_B_ receptors to LTP remain to be determined, the fact that both LTP and LTD of MNTB-LSO synapses critically depend on GABA signaling points to an important role of GABA co-release at these developing glycinergic synapses.

### Expression site of LTP

The expression of LTP was not accompanied by a change in the PPRs suggesting an unchanged probability of presynaptic vesicle release, which argues in favor of a postsynaptic expression of LTP. LTP resulted in an increase in pharmacologically isolated GlyR-mediated currents (Fig. 6). Due to the small amplitude of pharmacologically isolated GABA-mediated responses at this age and our experimental conditions, we were unable to reliably determine whether synaptic GABA responses also increased during LTP and thus cannot rule out a change in GABA_A_ receptor mediated currents, as is the case during LTD at these synapses^47^. However, because of the small amplitude of GABAA receptor-mediated currents and the fact that the amplitude of LTP of isolated GlyR mediated responses was similar to the amplitude of LTP under control conditions, any contribution of GABA_A_ receptor-mediated currents to the overall LTP would be minor. An increase in glycine receptor-mediated responses could result from an increase in the number of postsynaptic receptors and/or an increase in their open probability or single-channel conductance (Fig. 7). A rise in intracellular Ca^2+^ concentration and activation of calcium-/calmodulin-dependent protein kinase II (CaMKII) increases synaptic clustering of glycine receptors in Mauthner cells in fish^96^, which can result in larger quantal sizes of synaptic glycinergic currents^97^. Protein kinases regulate the strength of glycinergic synapses via phosphorylation of the glycine receptors itself^98–102^ or via glycine receptor anchoring protein gephyrin^99,103–105^. Due to the fast onset of LTP, modifications of existing synaptic glycine receptors likely underlies at least the early phases of LTP while gephyrin-mediated receptor clustering could play a more important role in later phases of LTP and the developmental increase of functional release sites in vivo^12^.

## Methods

### Animals and slice preparation

Mice of the strain C57BL/6J (Charles River) of either sex were used between postnatal days (P) 5–8. All animal procedures were performed in accordance with NIH guidelines and were approved by the IACUC of the University of Pittsburgh. Mice were deeply anaesthetized with isoflurane, decapitated and brains were removed and transferred to ACSF (composition in mM: NaCl 124, NaHCO_3_ 26, glucose 10, KCl 5, KH_2_PO_4_ 1.25, MgSO_4_ 1.3, CaCl_2_ 2, pH = 7.4, aerated with 95% O_2_/5% CO_2_). Coronal brain slices (300 µM thick), were cut using a vibrating microtome as previously described^23^. Slices containing both the LSO and MNTB were incubated for 1 h at 32 °C in an interface-style chamber and subsequently transferred to a recoding chamber where they were submerged and continuously perfused with aerated ACSF at room temperature throughout the recording session.

### Electrophysiology

Whole-cell patch clamp recording were made from borosilicate glass pipettes (tip resistances of 3–6 mΩ) and filled with internal solution containing in mM: 140 K-gluconate, 10 NaCl, 10 Hepes, 0.6 EGTA, 2 MgATP, 0.3 NaGTP and 2.5–10 phosphocreatine-Tris. When indicated, NaGTP was replaced by 2 GDP-β-s. If the internal solution contained 20 mM BAPTA, EGTA was omitted, K-gluconate lowered to 60 mM and 60 mM sucrose added. All internal solutions were pH adjusted using KOH to a final pH of 7.2–7.4 with an osmolarity of 280–290 mOsm.

The MNTB-LSO pathway was electrically stimulated (pulse duration 0.2 ms) using an ACSF filled pipetted (resistance ~ 1–3 mΩ), which was placed at the lateral edge of the MNTB. Paired-pulse stimuli with an interstimulus interval (ISI) of 50 ms were delivered at 0.1 Hz to elicit postsynaptic currents (PSCs). Stimulus strength was adjusted to evoke maximal responses^11^. Synaptic responses were recorded in voltage clamp (V_clamp_) at a holding potential of − 84 mV. This potential was chosen to increase the chloride driving force (calculated E_cl_—65.5 mV) and to minimize the activation of NMDA receptors by the co-release of glutamate from MNTB terminal^22,25^.

At least 20 successive stimuli were acquired during baseline (up to 15 min) after which recording were switched to current clamp (I = 0) and one of five induction stimulus protocols was delivered. Consecutive iterations (4 times) of induction protocols were triggered manually. They are described in the results and illustrated schematically in Figs. 1 and 2. The average series resistance (r_s_) was 14.9 ± 0.4 MΩ (n = 98) and was not compensated. Recordings with r_s >_25 mΩ or if r_s_ changed by more than 20% were excluded from analysis. Only cells with recordings ≥ 23 min post-induction stimulation were included in analysis.

### Statistical analysis

To assess the expression of LTP in individual neurons in response to stimulation paradigm or pharmacological treatment a minimum of 20 pre-stimulation measures (baseline amplitudes or baseline paired-pulse ratios) were compared to a minimum of 10 consecutive post-stimulation measures. Consecutive post-stimulation measures were taken starting at 2 min after the induction stimulation and every 7 min thereafter. Within-neuron pre- and post- stimulation measures were compared using 1-way ANOVA with Dunnett's multiple comparisons test. Neurons that maintained a significantly elevated amplitude at ~ 23 min post-stimulation (and during subsequent timepoints when obtained) were considered to express LTP.

For comparing response magnitudes within a group of cells amplitudes were normalized to the average pre-stimulation amplitude. Two-tailed paired t-test was used to compare average pre-stimulation responses (baseline) to the average of a minimum of 10 responses at ~ 23 min (23:59 ± 00:05 min) post induction. The means of normalized responses between two groups were compared using a two-tailed unpaired t-test. When comparing the means between more than 2 groups a 1-way ANOVA was performed. The normality of measures within a group was established using a Shapiro–Wilk test. One group (200 Hz Tetanic stimulus) was not normally distributed but was tested using a parametric test to maintain consistency across studies and to take a more conservative statistical approach. Testing the same dataset with a non-parametric test essentially yielded the same results. All results were considered statistically significant with a *p* < 0.05. Errors are reported as ± SEM.

## Data Availability

The datasets generated during and/or analyzed during the current study are available from the corresponding author on reasonable request.
