# Author Correction: Long-term potentiation of glycinergic synapses by semi-natural stimulation patterns during tonotopic map refinement

**DOI:** 10.1038/s41598-021-01422-z

**Published:** 2021-11-04

**Authors:** Eva C. Bach, Karl Kandler

**Affiliations:** 1 Departments of Neurobiology, Otolaryngology, and Bioengineering, University of Pittsburgh School of Medicine, Biomedical Science Tower 3, Rm. 10016, 3501 Fifth Avenue, Pittsburgh, PA 15261 USA; 2 Present Address: Department of Physiology and Pharmacology, Wake Forest School of Medicine, PTCRC, Rm. 230, 115 South Chestnut Street, Winston‑Salem, NC 27157 USA

Correction to: *Scientific Reports* 10.1038/s41598-020-73050-y, published online 9 October 2020

The Acknowledgements section in the original version of this Article contained an error.

“The authors thank Jongwon Lee for generating the data shown in Fig. 1A, Brian Brockway for general technical support, and Flora Antunes for valuable comments on a draft of the manuscript. This work was supported by DSF Charitable Foundation Grant-132RA03 (ECB) and NIDCD DC04199 (KK).”

now reads:

“The authors thank Jongwon Lee for generating the data shown in Fig. 1A, Brian Brockway for general technical support, and Flora Antunes for valuable comments on a draft of the manuscript. This work was supported by DSF Charitable Foundation Grant-132RA03 (ECB) and R01 DC004199 (KK).”

The original Article has been corrected.

